# Mechanical Properties of Porcine and Fish Skin-Based Collagen and Conjugated Collagen Fibers

**DOI:** 10.3390/polym13132151

**Published:** 2021-06-29

**Authors:** Hyunchul Ahn, Da Jeong Gong, Hyun Ho Lee, Joo Yeon Seo, Kyung-Mo Song, Su Jin Eom, Sang Young Yeo

**Affiliations:** 1 Advanced Textile R&D Department, Korea Institute of Industrial Technology, 143 Hanggaulro, Sangnok-gu, Ansan-si 15588, Korea; hahn@kitech.re.kr (H.A.); dajeong1656@kitech.re.kr (D.J.G.); 2 Fiber&Tech, 150, Jojeong-daero, Hanam-si 12930, Korea; simpleob@hotmail.com (H.H.L.); sirjooyeon@gmail.com (J.Y.S.); 3 Research Group of Food Processing, Korea Food Research Institute, 245 Nongsaengmyeong-ro, Wanju-gun 55365, Korea; rudah@kfri.re.kr (K.-M.S.); Eom.Su-jin@kfri.re.kr (S.J.E.)

**Keywords:** collagen fiber, wet-spinning, spinnability, conjugated fiber, mechanical properties

## Abstract

Collagen is a protein that is a major component of animal skins and tendons. It is used in various medical, cosmetic, and food products through extraction and purification. The fibrous products of purified collagen fibers extracted from raw mammal materials have relatively excellent mechanical properties and are used for high-end medical products. In this study, we examined collagen materials produced from porcine and fish skins, which are major sources of collagen raw materials. We examined a method for spinning collagen fibers from fish skin-based collagen and analyzed the physical properties of those collagen fibers. In addition, we examined the characteristics and advantages of conjugated fibers according to their porcine- and/or fish skin-based compositions. The spinnability and mechanical properties of these conjugated fibers were analyzed according to their compositions. The mechanical properties of collagen structure are determined by hydroxyproline content and can be manipulated by the composition of collagen in the conjugated fibers.

## 1. Introduction

Collagen is a natural protein that is the primary material in animals and is abundant in skin, tendons, cartilage, and bones. There are several types of collagen, categorized by region and role. The most common is Type-I collagen, a structural protein prevalent in skin [1]. The unique triple helical structure of collagen consists of two α1 chains and one α2 chain, which form an amino acid array in the form of glycine-X-Y such as proline and hydroxyproline [2]. Collagen is perfectly biocompatible and is widely used as a medical and cosmetic material. In particular, it is used in various surgical materials and cell research as a biopolymer that can be used both inside and outside the human body [3]. Currently, commercial collagen is mainly extracted from mammal skins and organs [4]. In particular, collagen products in the form of fibers and structures are produced using collagen extracted from mammals with a relatively high mechanical behavior. Mammal-skin-based Type-I natural collagen fibers generally have an elastic modulus of 0.3 to 1.2 GPa and an elongation of approximately 10%. For dehydrated collagen fibrils, a modulus of 1.8 to 2.25 GPa was observed due to higher molecular packing [5]. These mechanical properties were attributed to the amino acid sequence and the coiling of a collagen fibril with a particular triple-helical structure [6]. In addition, some collagen properties may be lost during the purification process. After purification, however, collagen is widely used for medical purposes such as drug delivery, wound dressing, nerve regeneration, and eye regeneration. Additionally, collagen has been used in various studies because of its typical physical and chemical characteristics and excellent biocompatibility [7]. Recently, however, the supply of collagen extracted from mammals has been limited and is mainly obtained from bovine and porcine tendons and skins. This has led to problems including infectious diseases, environmental concerns, increased allergies, and has also been problematic for religious reasons [8]. As a result, collagen has recently been extracted from more diverse sources, such as fish and plants [9]. Recently, a large amount of collagen has been collected from fish byproducts, such as skin, scales, and bones [10]. The yield of collagen extracted from fish skin is high, which has both improved the value of fish byproducts and reduced environmental problems [11]. Recently, low-molecular-unit collagen extracted from fish skin has been used in cosmetic and food collagen products. However, such collagen has thermal and mechanical limitations, and is, therefore, not used in medical applications [12].

The collagen fibers used in medical applications are mainly manufactured using collagen extracted and purified from mammals. Various studies have been conducted on the formation of collagen structures through nano-web manufacturing using electrospinning as well as fibers through wet spinning [13,14,15]. Various studies on the structural characteristics and physical properties of collagen have been carried out over the past several decades [16,17]. Amino acid compositions are known to vary depending on the species and environment, even when extracted from Type-I collagen, and these variations produce varying physical properties [18,19]. In particular, mechanical and thermal properties depend greatly on the hydroxyproline content in the collagen structure [20]. Hydroxyproline facilitates intermolecular binding through the hydrogen bonding in the triple helical structure in collagen. Therefore, hydroxyproline content improves physical stability [21]. As a result, the hydroxyproline content is higher in mammals with hard skins or species living at high temperature environments. Fish have a relatively lower hydroxyproline content than that in land animals, particularly, species that live in the deep ocean [22]. Recently, studies have analyzed various fish-based collagen extractions, and most fish species from which high amounts of collagen can be extracted have been analyzed [23]. However, fish skins are affected not only by species, but also by the living environments and ages of individual fish. This produces large and disadvantageous deviations, because the circulation of epidermal cells and structures is faster than it is in terrestrial animals [24].

**Figure 1:**
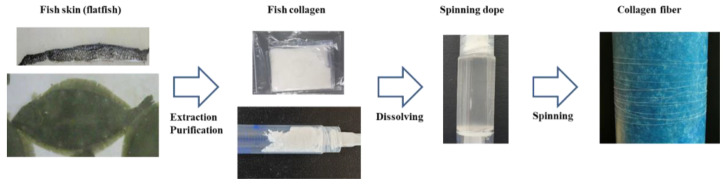
Schematic representation of fish skin-based collagen extraction and fiber spinning.

In this study, the fish skin-based collagen was extracted and purified to analyze its material properties (Figure 1). The properties of the extracted fish skin collagen were analyzed and the possibility of using it as collagen fiber was examined. The amino acid composition and thermal characteristics of porcine collagen and fish skin collagen were analyzed, and collagen fiber spinnability was assessed for each material. The mechanical properties of the wet-spun collagen fibers were examined, the material properties were analyzed, and the possibility of complex collagen material spinnability was examined. Moreover, the preparation and physical properties of the conjugated collagen fibers were investigated. Based on this research, we analyzed the spinning conditions and potential uses of the conjugated collagen fibers.

## 2. Materials and Methods

### 2.1. Materials

High-purity powdered collagen refined for medical purposes was used as the commercial product (Theracol^®^, Athena, Korea) in the study and it is porcine skin-based collagen. The fish collagen was extracted and purified from flatfish (Paralichthys olivaceus) [25] and tilapia (Oreochromis mossambicus) [26]; these species were cultivated in Korea. A typical collagen extraction method was used [4,27]. In short, collagen was extracted from pretreated raw fish skin materials at low temperatures through ultrasonic extraction, centrifugation, and freeze drying, and purified through salt precipitation and ion exchange chromatography. The final purified collagen was used in a freeze-dried state, and the dope was prepared by dissolving it in acid. Acetic acid (EP, 99.5%, Daejung, Siheung, Korea) was used for the preparation of dope for wet-spinning, and acetone (LP, 99.98%, SK Chemical, Seoul, Korea) was used as the coagulation solvent.

### 2.2. Material Analysis

Collagen samples were hydrolyzed with 6 N HCl at 120 °C for 4 h for amino acid composition analysis. The amino acid composition was detected by HPLC (UPLC 3000, Thermo Fisher, Somerset, MD, USA) using an AccQ-Tag amino acid analysis column (particle size 4 μm, length 150 mm, internal diameter 3.9 mm). Eluents were AccQ-Tag eluent (A) (Waters Co., Milford, MA, USA), acetonitrile (B), and water (C) (J. T. Baker Chemical Co., Radnor, PA, USA). The gradient conditions were as follows: 100% A initial, 99% A and 1% B over 0.5 min; 95% A and 5% B over 18 min; 91% A and 9% B over 19 min; 83% A and 17% B over 28 min; 60% B and 40% C over 35 min; and 100% A over 38 min. The flow rate was 1.0 mL/min, and the detection wavelength was 254 nm. The column oven temperature was maintained at 37 °C. The sample and amino acids were de-rivatized before injection using an AccQ-Tag Ultra derivatization kit (Waters Co., MA, USA) [28]. Contents of each amino acid were expressed as the mean ±SD (*n* = 2) and statistical significance (*p* < 0.05) was determined by one-way analysis of variance (ANOVA) and Duncan’s post hoc test using SPSS 20 software (IBM, Armonk, NY, USA).

Enzyme digestion tests and differential scanning calorimetry (DSC) were used to measure the denaturation temperature of the collagen. For the enzyme digestion tests, the samples were treated at 20, 25, 30, 35, 40, 45, 50, and 95 °C for 30 min using a heating block (Thermo Fisher Scientific, Waltham, MA, USA) and digested using 0.5% pepsin at 15 °C for 1 h. The samples were then analyzed by sodium dodecyl sulfate-polyacrylamide gel electrophoresis (SDS-PAGE) with 7.5% precast protein gels (Bio-Rad, Hercules, CA, USA). Pre-stained protein standards (10–250 kDa) were used as size markers.

A 2 mg collagen sample was sufficiently dissolved in 0.05 M acetic acid for DSC analysis (*n* = 3 for each sample). The DSC 4000 system (Perkin Elmer, Waltham, MA, USA) was used for an analysis from 20 to 100 °C at 10 °C/min under nitrogen [29].

### 2.3. Fiber Spinning

**Figure 2:**
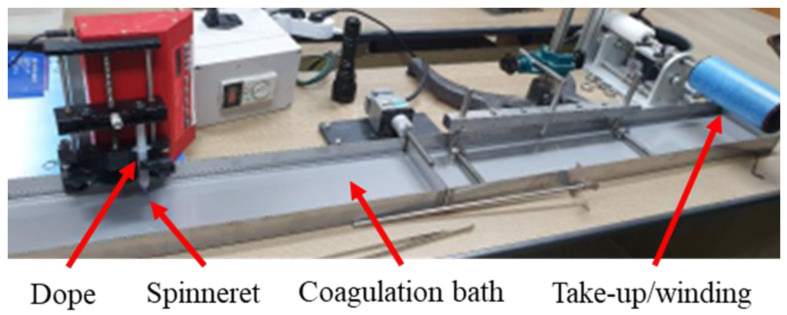
Lab-scale equipment of collagen fiber spinning.

Collagen fiber spinning was performed using lab-scale wet-spinning equipment according to the methods used to manufacture collagen fibers for medical purposes [30]. A schematic of the collagen fiber spinning is shown in Figure 2. The dope was prepared by dissolving collagen in 0.5 M acetic acid, with a 5 wt% collagen concentration. For the mixed porcine and fish collagen, two materials were mixed in the powder state and then dissolved in acid. A low-temperature process is required because of the characteristics of collagen materials. The dissolution was performed at 4500 rpm at 15 °C using a low-temperature centrifuge for collagen mixing and dope de-foaming. The spinning process for the manufactured dope was performed at a rate of 0.001–0.002 mL/min using a syringe pump, and the spinneret was a single nozzle of the 30 Gauge nozzle. Fiber spinning was performed in an acetone solvent coagulation bath with a fiber winding speed of 0.33 m/min. The overall spinning process was carried out at room temperature (25 °C). After coagulation and fiber formation, there was no additional washing process during the spinning. The collagen fibers were washed and dried shortly before mechanical testing. The types of collagen materials used in this study were porcine, flatfish, and tilapia singe composition collagen, and porcine/tilapia conjugated collagen. The fibers were not treated such as cross-linking after spinning process.

### 2.4. Mechanical Analysis

The wet-spun fibers were observed by electron microscopy (FE-SEM, SU8000, Hitachi, Tokyo, Japan), and fiber diameters were measured from the corresponding images. The mechanical properties were measured by a single fiber tensile test (FAVIMAT, Textetchno, Mönchengladbach, Germany), and tested at a speed of 20 mm/min at a gauge length of 20 mm. Fiber tensile strength and linear density were measured using FAVIMAT tensile tests. More than 10 samples were tested for each case.

## 3. Results and Discussions

### 3.1. Material Analysis

**Figure 3:**
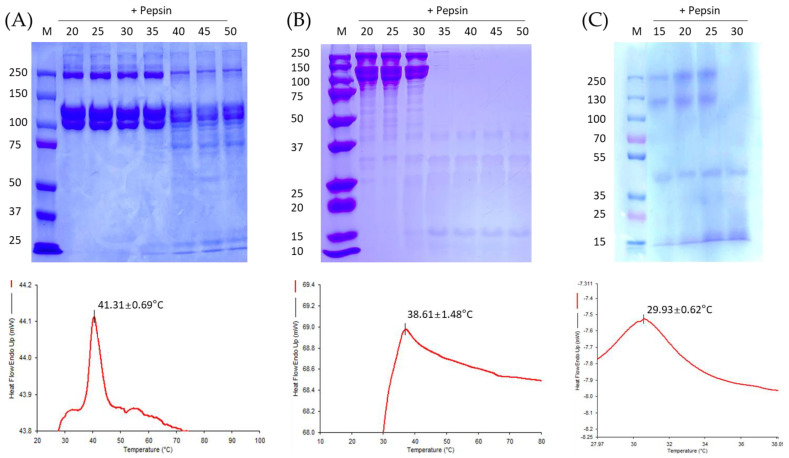
Thermal stability of collagen measured by enzyme digestion and DSC analysis. (**A**) porcine, (**B**) tilapia and (**C**) flatfish skin-based collagen.

Amino acid analysis and collagen analysis showed that most of the amino acids contained in the collagen were Gly, Hyp, Ala, and Pro. The Hyp contents for porcine, tilapia, and flatfish were 13.3 ± 0.3%, 12.7 ± 0.2%, and 10.4 ± 1.0%, respectively (Table 1). Similar to a previous study [22], the hydroxyproline content was low in collagen extracted from fish, and varied between fish species. The proline and hydroxyproline contents were lower for flatfish, which live in relatively cold-water environments compared to tilapia, which is cultivated in warm water. However, overall, porcine collagen contained more of the amino acid content that determines the thermal and mechanical properties. Acid-soluble collagen is not decomposed by proteases such as pepsin (except collagenase). However, protease can decompose gelatin, which is the denaturing material for collagen [14]. Using this characteristic, the denaturation temperature of collagen was detected by an enzyme digestion analysis. As shown in Figure 3, for temperatures at which collagen is denatured, low molecular bands are observed rather than high molecular bands. The porcine and tilapia collagen appeared to be stable for at least 30 min below 35 °C, 30 °C, and 25 °C. As heat transforms collagen into gelatin, the triple helix is released and the heat flow end-up value rises [20]. According to the DSC analysis, the melting temperatures of porcine, tilapia, and flat fish collagen were 41.31 ± 0.69 °C, 38.61 ± 1.48 °C, and 29.93 ± 0.62°C, respectively, similar to the results of SDS-PAGE. Flatfish skin-based collagen had lower thermal stability than collagen from tilapia. In this study, the thermal characteristics of porcine and tilapia skin-based collagen were compared and analyzed, because the conjugated fibers were manufactured based on these materials. Furthermore, as a result of the DSC analysis, even the fish skin-based collagen, flatfish showed significantly low thermal stability, and tilapia collagen showed a high value similar to that of porcine collagen. As it is known, since Hyp content has the greatest effect on thermal stability, it is considered to show such thermal properties according to the Hyp content as the material used in this study. However, the melting point showed a result proportional to the Hyp content as described above, but the behavior according to temperature was different, which is thought to be due to the difference in the composition of porcine and other fish skin-based collagen. In addition, since Hyp content has a large influence on mechanical properties, it is thought to show such tendency.

### 3.2. Structure and Mechanical Properties

**Figure 4:**
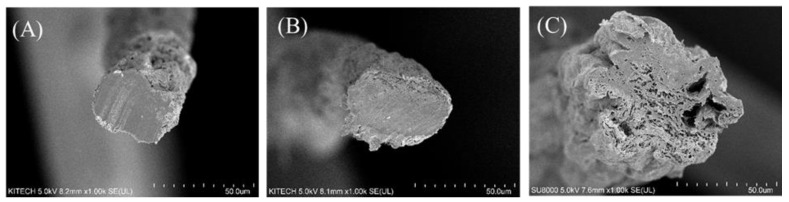
Cross section of single composition collagen fibers. (**A**) porcine, (**B**) tilapia skin-based, and (**C**) flatfish-skin-based collagen. The scale of all figures is 50.0 μm.

**Figure 5:**
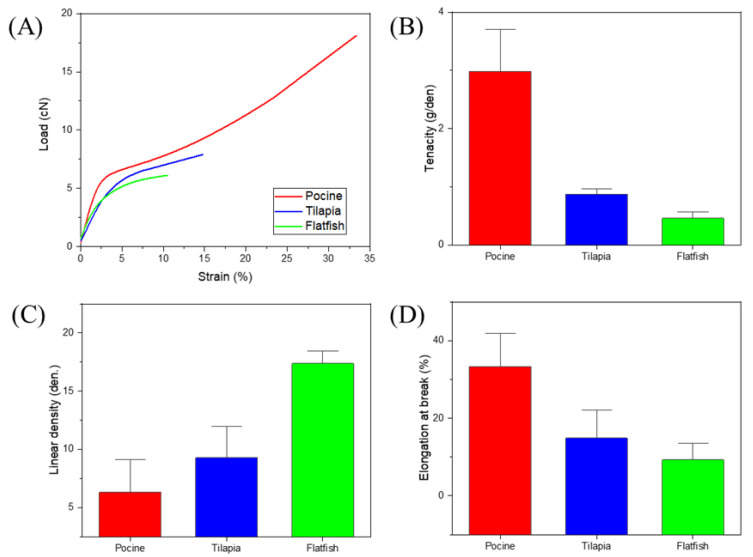
Mechanical properties of porcine, tilapia, and flatfish single composition collagen fibers. (**A**) typical load-strain behavior for each fiber, (**B**) tenacity of each fiber, (**C**) linear density of each fiber, and (**D**) elongation at break of each fiber. At least 20 fibers were analyzed for each case with standard deviation.

First, a cross section of each fiber manufactured by the wet-spun process was examined, and the mechanical properties of the fiber were measured. As mentioned above, we found that because of its amino acid composition and resulting mechanical properties, fish skin-based collagen was much more difficult to wet-spin than porcine collagen. This was particularly true for collagen extracted from flatfish, a cold-water species with the worst spinnability and lowest uniformity. As shown in Figure 4, a low density and uneven cross section can be seen for the same spinning conditions. According to the amino acid composition, the mechanical properties were relatively low because the hydroxyl amino acid content was relatively low, and the coagulation behavior was slow in the coagulation bath. However, the fiber density was similar to that of the tilapia skin-based collagen, indicating that the finer fiber was produced in the same spinning process. The mechanical properties of each fiber are shown in Figure 5. According to the tensile test of fibers, the tenacity of porcine, tilapia, and flat fish collagen were 2.98 ± 0.41, 0.88 ± 0.07, and 0.46 ± 0.16 g/den, respectively. The tilapia skin-based collagen had a 30% higher linear density and 30% lower strength than the porcine collagen. The flatfish skin-based collagen fibers had a very high linear density and low mechanical properties; its structure showed low elongation and strength due caused by low density and many pores (which act as defects). The tilapia skin-based collagen fiber had a similar cross-section to the porcine collagen fiber, but its tensile behavior was similar to that of flatfish skin-based collagen. This indicates that the spinnability of the tilapia skin-based collagen fibers was lower than for the porcine collagen, and that many micro pores are present in the spun fiber. However, the initial stiffness was very similar to that of the other three fibers. The initial behavior is based on the internal molecular structure, which is the result of amino acid bonding rather than hydrogen bonding [21]. However, it is considered that strength differences are caused by the differences in hydrogen bonds between molecules and the molecular density. Moreover, in contrast with flatfish skin, it is possible to produce collagen fibers from tilapia skin, because wet-spinning processes are feasible and procedures are uniform. Therefore, in this study, we compared porcine collagen and tilapia skin-based collagen and examined the mechanical properties of their conjugated collagen fibers.

**Figure 6:**
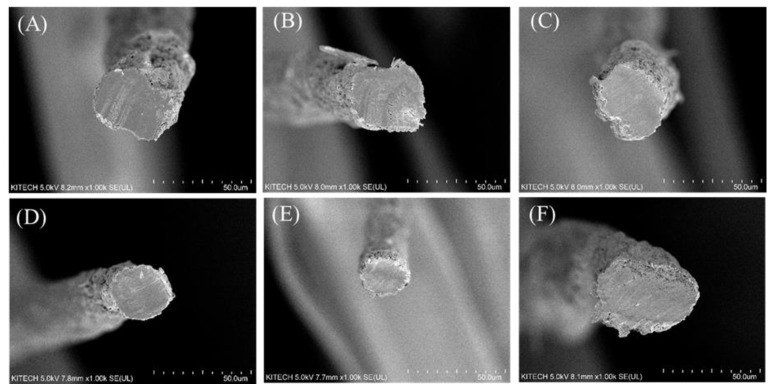
Cross section of porcine/tilapia conjugated collagen fibers. (**A**) Porcine collagen 100% fiber, (**B**) porcine collagen 80% fiber, (**C**) porcine collagen 60% fiber, (**D**) porcine collagen 40% fiber, (**E**) porcine collagen 20% fiber, and (**F**) tilapia collagen 100% fiber. The scale of all figures is 50.0 μm.

**Figure 7:**
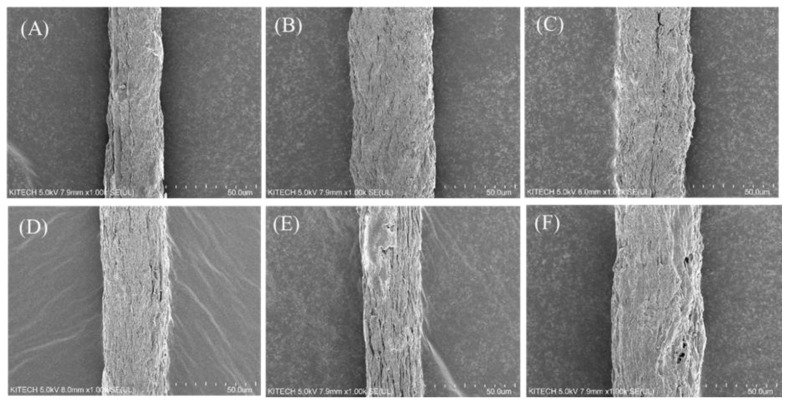
Surface and diameter of porcine/tilapia conjugated collagen fibers. (**A**) Porcine collagen 100% fiber with 31.3 (3.69) μm averaged diameter, (**B**) porcine collagen 80% fiber with 37.3 (4.04) μm averaged diameter, (**C**) porcine collagen 60% fiber with 39.3 (4.32) μm averaged diameter, (**D**) porcine collagen 40% fiber with 35.7 (3.56) μm averaged diameter, (**E**) porcine collagen 20% fiber with 31.5 (3.05) μm averaged diameter, and (**F**) tilapia collagen 100% fiber with 50.1 (4.23) μm averaged diameter. The scale of all figures is 50.0 μm. At least 5 fibers were analyzed for each case with standard deviation.

Six samples of conjugated collagen fibers from porcine and tilapia skins were prepared, with varying fractions of each material. The porcine collagen contents were 100, 80, 60, 40, 20, and 0%. According to the SEM images shown in Figure 6 and Figure 7, there were no significant differences in the cross-sectional and surface structures of the fibers. As mentioned above, tilapia skin-based collagen fibers tend to be low in density and thick in diameter, but the conjugated fibers, as Figure 7, basically show a diameter difference within 20%. Due to the spinning process of this research and without cross-linking, there is a deviation of about 10% each fiber, so the difference in spinnability and diameter due to the composition of collagens seems to not be significant. Among the conjugated fiber, the 20% porcine collagen fiber had the smallest diameter, but this seems to have been due to the fiber manufacturing environment. Additionally, it fell within the deviation range of the single-nozzle wet-spun fibers without pre- and post-treatment under the current lab-scale spinning system. In addition, the fiber diameters and linear densities showed large deviations, but the deviations in tenacity (which will be discussed in the next) were significantly reduced. Therefore, the internal structure appeared to be highly uniform. Moreover, although non-uniformity and distributions occurred in the simple fiber spinning process, the spinnability was sufficiently secured and the fiber shape was sufficiently uniform for conjugated fiber spinning to be performed without any other processes. It means the conjugated fiber has no significant post-treatment problems such as cross-linking. In addition, as seen in Figure 7, the modifications in the tilapia skin-based collagen produced rough surfaces, which seems to have been due to the characteristics of the fish-skin-based collagen, as shown by the flatfish skin-based collagen fiber. The coagulation process was slower and less stable than that for porcine collagen, which reduced the density and decreased the mechanical properties based on the molecular bonding between hydroxyl amino acids. However, because of their low density and porous structures, these materials have relatively high hydrophilicity, and water absorption is expected to improve. In conjugated collagen fibers, the advantages of both porcine and fish skin-based materials can be maximized by using a combination of both.

**Figure 8:**
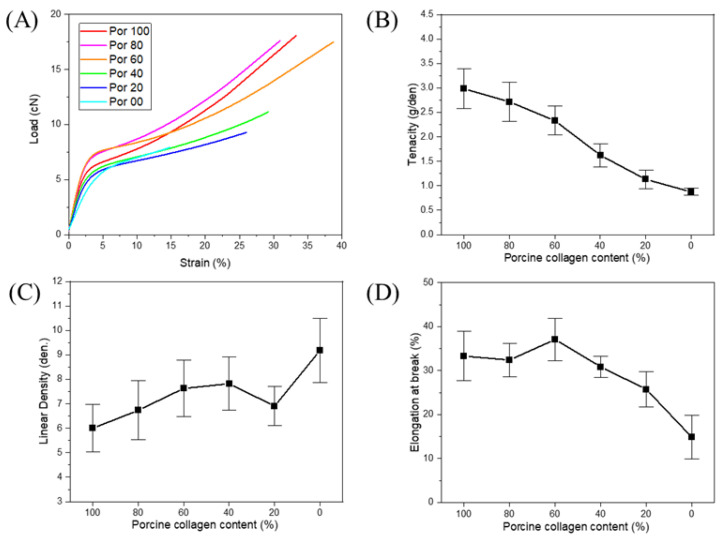
Mechanical properties of porcine and tilapia conjugated collagen fibers. (**A**) typical load-strain behavior of each fiber, (**B**) tenacity of each fiber, (**C**) linear density of each fiber, and (**D**) elongation at break of each fiber. At least 20 fibers were analyzed for each case with standard deviation.

The results for the mechanical properties are shown in Figure 8. No chemical reactions were observed in the conjugated fibers, indicating that the proportional results were based on the collagen content. However, the linear densities were similar, with differences in elongation. Overall, the figure shows the results of two phases, because porcine collagen plays a dominant role in the mechanical properties. Therefore, the strengths of the conjugated collagen fibers varied according to changes in the collagen content, for either high porcine content or high fish skin-based content. The differences in strength were largest between the samples having 40% and 60% porcine collagen content. The Figure 8B shows that the same tendency when the porcine collagen is dominant in the representative tensile behavior and the fish skin-based one is dominant. As shown in Figure 8B, the tenacity of the conjugated fibers is generally reduced in proportion to the collagen composition ratio, and the values are reduced by 13, 18, 34, 23, and 12%, respectively. For porcine collagen, more hardening behavior can be observed after the yield point, but the effects were small for fish skin-based collagen.

In this study, we found that the mechanical properties of collagen structure are determined by hydroxylproline content. In contrast, tensile behavior is determined both by hydroxylproline and by various amino acid interactions and fiber microstructures. In addition, because fibers dominated by porcine collagen and fibers dominated by fish skin-based collagen behave differently, this research can be applied to the production of structures using various collagen conjugated fibers and the production of fiber-based composite structures and scaffolds. For example, in the case of collagen fiber applications requiring excellent mechanical properties, conjugated collagen fibers with mechanical behavior similar to that of porcine collagen fibers can be produced using only a small skin collagen content, and the increased hydrophilic properties and structures can be controlled. Especially, it is not easy to control the properties of medical collagen fibers such as sutures and lifting yarns because it is not easy to add other substances other than collagen. However, it is thought that the conjugated fibers can be manufactured through properties and behavior control according to collagen composition.

## 4. Conclusions

Collagen fibers have different mechanical properties depending on their amino acid compositions, which vary according to the raw material from which the collagen was extracted. Porcine collagen has better mechanical properties than fish skin-based collagen. These properties can be determined from the hydroxyproline content, through amino acid analysis. In addition, the fiber spinnability of the porcine and fish skin-based collagen materials was analyzed, and conjugated collagen fibers were manufactured to manipulate the mechanical properties of the wet-spun collagen fibers, maintaining the respective advantages of porcine and fish skin-based collagen. This effect can be more accurately identified through the microstructure analysis in the future. It is expected that by utilizing fish skin-based collagen, optimized collagen fibers can be produced in collagen structures requiring optimized mechanical properties. Furthermore, fish skin-based collagen can be used in high-end medical products that currently use only mammal-based collagen.

## Figures and Tables

**Table 1:** Amino acid composition of collagen.

Amino Acid	Porcine	Tilapia	Flatfish
Gly	35.3 ± 0.3 ^b^	34.5 ± 0.2 ^b^	36.8 ± 0.5 ^a^
Hyp	13.3 ± 0.3 ^a^	12.7 ± 0.2 ^a^	10.4 ± 1.0 ^b^
Pro	12.8 ± 0.0 ^a^	12.0 ± 0.1 ^b^	11.6 ± 0.2 ^b^
Ala	11.1 ± 0.1 ^b^	11.8 ± 0.1 ^b^	13.9 ± 0.4 ^a^
Arg	7.9 ± 0.0 ^b^	7.2 ± 0.1 ^c^	8.2 ± 0.1 ^a^
His	3.8 ± 0.2 ^a^	4.1 ± 0.0 ^a^	2.1 ± 0.0 ^b^
Ser	2.7 ± 0.2 ^a^	2.7 ± 0.0 ^a^	2.0 ± 0.0 ^b^
Tyr	2.7 ± 0.2 ^b^	2.1 ± 0.0 ^c^	3.1 ± 0.1 ^a^
Glu	2.1 ± 0.2 ^b^	3.1 ± 0.1 ^a^	1.4 ± 0.1 ^c^
Leu	2.1 ± 0.1 ^a^	2.1 ± 0.1 ^a^	1.9 ± 0.1 ^a^
Lys	2.0 ± 0.0 ^a^	1.6 ± 0.0 ^c^	1.8 ± 0.0 ^b^
Thr	1.3 ± 0.0 ^c^	2.2 ± 0.0 ^a^	2.1 ± 0.0 ^b^
Phe	1.0 ± 0.0 ^b^	1.0 ± 0.1 ^ab^	1.2 ± 0.1 ^a^
Asp	0.7 ± 0.0 ^b^	0.9 ± 0.0 ^a^	0.9 ± 0.0 ^a^
Ile	0.7 ± 0.0 ^a^	0.8 ± 0.0 ^a^	0.6 ± 0.0 ^a^
Cys	0.3 ± 0.4 ^a^	0.8 ± 0.0 ^a^	0.6 ± 0.0 ^a^
Met	0.2 ± 0.2 ^b^	0.4 ± 0.0 ^b^	1.2 ± 0.1 ^a^
Val	0.0 ± 0.0 ^b^	0.1 ± 0.0 ^a^	0.0 ± 0.0 ^b^

a–c represented significant difference (*p* < 0.05).

## Data Availability

Not applicable.
